# Joining the dots: Conditional pass and programmatic assessment enhances recognition of problems with professionalism and factors hampering student progress

**DOI:** 10.1186/1472-6920-11-29

**Published:** 2011-06-07

**Authors:** Tim J Wilkinson, Mike J Tweed, Tony G Egan, Anthony N Ali, Jan M McKenzie, MaryLeigh Moore, Joy R Rudland

**Affiliations:** 1 University of Otago, Christchurch, C/- The Princess Margaret Hospital, PO Box 800, Christchurch, New Zealand; 2 Medical Education Unit, University of Otago, Wellington, PO Box 7343, Wellington 6242, New Zealand; 3 Faculty Education Unit, Faculty of Medicine, University of Otago, PO Box 56, Dunedin 9054, New Zealand; 4 Medical Education Unit, University of Otago, Christchurch, PO Box 4345, Christchurch 8140, New Zealand

**Keywords:** Assessment, Professionalism, Monitoring progress, Feedback

## Abstract

**Background:**

Programmatic assessment that looks across a whole year may contribute to better decisions compared with those made from isolated assessments alone. The aim of this study is to describe and evaluate a programmatic system to handle student assessment results that is aligned not only with learning and remediation, but also with defensibility. The key components are standards based assessments, use of "Conditional Pass", and regular progress meetings.

**Methods:**

The new assessment system is described. The evaluation is based on years 4-6 of a 6-year medical course. The types of concerns staff had about students were clustered into themes alongside any interventions and outcomes for the students concerned. The likelihoods of passing the year according to type of problem were compared before and after phasing in of the new assessment system.

**Results:**

The new system was phased in over four years. In the fourth year of implementation 701 students had 3539 assessment results, of which 4.1% were Conditional Pass. More in-depth analysis for 1516 results available from 447 students revealed the odds ratio (95% confidence intervals) for failure was highest for students with problems identified in more than one part of the course (18.8 (7.7-46.2) p < 0.0001) or with problems with professionalism (17.2 (9.1-33.3) p < 0.0001). The odds ratio for failure was lowest for problems with assignments (0.7 (0.1-5.2) NS). Compared with the previous system, more students failed the year under the new system on the basis of performance during the year (20 or 4.5% compared with four or 1.1% under the previous system (p < 0.01)).

**Conclusions:**

The new system detects more students in difficulty and has resulted in less "failure to fail". The requirement to state conditions required to pass has contributed to a paper trail that should improve defensibility. Most importantly it has helped detect and act on some of the more difficult areas to assess such as professionalism.

## Background

There has been much progress in the assessment of clinical competence and performance. Recognition that many assessment tools were unreliable resulted in a quest for, and changes to, more reliable ones. From such moves arose a threat to validity as the drive for objectivity was often replaced by objectification to the point of trivialisation of the assessment task [1]. Evidence that reliability arises more from the aggregation of assessments, rather than from the over-specification of criteria led to the rise of more authentic assessment tools, often in the workplace. As a consequence we can take a more programmatic approach to assessment whereby a variety of tools in a variety of settings lead to both enhanced reliability and validity [2]. Our next challenge is to consider how to create a system that uses seemingly disparate pieces of information to inform defensible decisions. Added to this is the complexity of so called "sub threshold" concerns where a candidate may cause some concern on a number of assessments but none, on its own, is sufficient to trigger action [3]. However, taken in their entirety such assessment results suggest a pattern of performance that should be acted on. Regrettably patterns like these are often seen in retrospect, sometimes after the opportunity for action has passed. There is a need therefore for research into ways to improve the quality of assessment systems, not just assessment tools [4].

"Failure to fail", particularly during clinical attachments or clerkships, has been well described [5]. Some evidence suggests that the final decision on a student's progress is not always consistent with an attachment supervisor's overall judgement of performance [5-8]. One informal survey suggested over 50% of faculty members indicated they passed students who they felt should fail [9]. Three quarters of faculty surveyed in ten US medical schools rated "unwillingness to record negative evaluations" as a problem [5,10]. It has been reported that concerns about underperformance in medical students are often not recorded formally [11] and clinical assessments do not always accurately reflect student performance [8,11]. Such students are at risk of becoming incompetent doctors [11,12].

When there is uncertainty around a student's level of competence, some apply different terms such as borderline, bare fail, bare pass, or "needs assistance" [13]. We previously used the term borderline but found this tended to defer the problem; at the end of a year a final decision would have to be made and on review a student might be seen to have a number of "borderlines" and there was little information on whether the student had met the required standards or not. Worse still, there was minimal documentation so a fail, based on several "borderlines" and some "passes", was not really possible because of the lack of defensible evidence. This lack of evidence made it unclear whether several "borderlines" reflected a generic problem seen throughout the programme or reflected a range of different problems. Consequently, borderline tended to be used more when there was assessor uncertainty rather than when a student only just fell on one side of the pass-fail threshold. This use of a borderline grade required no attempt to identify or specify the nature of the uncertainty or of the student's poor performance.

A programmatic approach to assessment facilitates validity and reliability of decisions through triangulation of data, and allows weaknesses of some assessment tools to be countered by strengths of others. Suggestions for some features of such a system include:

### 1. Transparency

Clearly and explicitly articulate a longitudinal, integrated, and shared assessment programme which is vetted by faculty and about which students are informed at the beginning of, and periodically throughout, their programme of study. This will address any concerns about legal liability [3].

### 2. Avoid compensation between disparate attributes

Rather than giving single ratings for a whole attachment, define the subcomponents, define the expectations and report on these subcomponents as well as an overall rating for the whole attachment [6,7,14]

### 3. Direct observation

Ensure enough formal assessments include direct observation. Assessments that are independent, varied, contextual and valid can contribute to a cumulative performance profile [3,15,16].

### 4. Make decisions based on accumulated evidence

Develop a mechanism whereby an accumulation of coherent evidence is provided rather than rely on disparate small pieces of evidence. When there is doubt about a student's achievements, obtain more evidence to enable a decision [2,17-19].

### 5. Use multiple reviewers and qualitative data

Assemble an independent panel of reviewers who make decisions on progress, through a progress committee, based on descriptive qualitative information provided by supervisors [2,3,6,20-22]. Such qualitative evaluations of students should describe specific behaviours and issues, not generalised and vague judgements [3].

### 6. Conditional promotion

Make "conditional promotion" decisions rather than a series of marginal passes which can be too vague to prompt action [2,3,17].

### 7. Feedback

Ensure there is an on-going feedback mechanism in place so that any final decision does not come as a total surprise to the learner [2].

### 8. Feedforward

Problems that are identified should be shared with the faculty who subsequently teach the students [20,22]. It should be noted that attachment supervisors are divided about this. A recent US survey revealed that only 14% of institutions have written policies about sharing information and 57% of clerkship directors design remediation plans for struggling students [20]. There is an argument that a student's learning can be enhanced if subsequent teachers can respond to and build on a student's known learning needs. Furthermore, our duty to the public to ensure competent graduates outweighs any disadvantage to the student. However the counterargument is that sharing information regarding struggling students has the potential for creating both negative and positive biases toward the student identified as such and can create a "self-fulfilling prophecy" [23]. This may be more likely to occur if there are no formalised assessments of competence other than global ratings by supervisors, who have conflicting roles as trainer and assessor [24].

We have developed and implemented a new system that acts on all of these recommendations. Furthermore, we now have outcomes from that system that we can compare with our previous system. The aim of this study is to describe this system, developed within a medical course, and to report on its progress and effects.

## Methods

### Context

In the last three years of our six-year course, students rotate through a series of 5-8 week clinical attachments (block modules). Alongside these is a coordinated backbone of vertical modules of learning relating to less context specific areas such as pathology, pharmacology and medical ethics. Each of the clinical attachments and most of the vertical modules conduct in-course summative assessments which contribute to deciding at the end of year whether a student is permitted to proceed to the next year. In the case of year 5, these in-course assessments contribute to deciding whether a student is permitted to sit the end of year major high stakes summative assessment that assesses knowledge and its application in written and multiple choice question examinations, and consultation abilities in an Objective Structured Clinical Examination [25]. Students can therefore be prevented from proceeding from year 4 to year 5, or from year 6 to graduation by not passing the in-course assessments. Progression from year 5 to year 6 requires passing the in-course assessments as well as passing the end of year examinations.

### Previous assessment system

In the past, student achievement was determined by aggregating results of formal assessments (e.g. of knowledge or observed clinical skills) and supervisor opinions into a grade or percentage for each clinical attachment. This aggregation could then result in one area of weakness (e.g. professionalism) being compensated by another area of strength (e.g. knowledge). Furthermore, it was very difficult to fail someone if they comfortably passed on attributes that could easily be represented by a quantitative numerical measure if they underperformed on less easily measurable attributes. In addition, borderline grades that were seen across a number of attachments, and that collectively raised cause for concern, could not easily be acted on in making progress decisions.

Since then we have devised, implemented and monitored a system to detect poorly performing students, including where there are concerns about professionalism [26]. This enables action to be taken in relation to expressed concerns and is followed by monitoring. The outcomes of this then inform decisions about student progress. Over the past four years, it has operated in the latter three clinical years of our course but is now also being implemented in the earlier stages of the course. It draws on many of the elements suggested by others, and combines them into one system.

### New assessment system

The revised assessment system is built on four foundations:

1. The use of "Conditional Pass" (CP). This term evolved from "needs assistance" or "borderline" (used in the old system) where students may have barely passed or failed an assessment or where there was uncertainty about a student's true ability [27]. We found the use of the term "borderline" created ambiguity for both staff and students, resulting in staff using the term in a range of situations: when the decision was difficult, if there was a paucity of data, or if there was uncertainty about the validity of the assessments. In contrast, when CP is awarded, it requires the module convenor to identify the nature and, where possible, cause of the concern and, even where there is still doubt about these, to state specifically the conditions that need to be met for the student to pass. Some examples of situations where a CP might be awarded are:

• Not reaching the required standard on a single assessment (such as an assignment or OSCE) in which case the condition would be either to repeat that assessment and achieve the required standard or to demonstrate in subsequent modules that the required standard in that attribute has been met.

• Failure to meet deadlines, in which case the condition would be to demonstrate reliability in meeting deadlines in subsequent modules.

• Poor attendance and unreliability, in which case the condition would be to demonstrate appropriate behaviours in subsequent modules.

**Figure 1:**
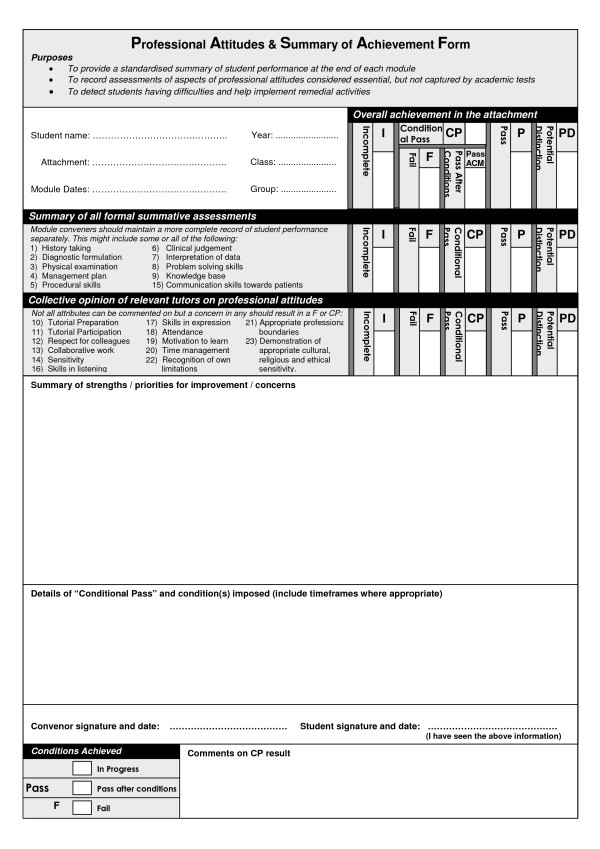
**Reporting form completed at the end of each module**.

Although each module could have several assessments, failure to achieve the standards in any summative assessment leads to a CP for that module. In each case, the concerns are documented and made known to the student, initially by the module convenor at the end of that module. The conditions help to clarify the concern and identify where relevant information is lacking. Most importantly, the conditions make clear to the student what needs to be achieved. In most cases, the convenor of the next module is also informed (feedforward [23]). Assessment results of student performance, including professional attributes, are summarised and recorded within a form that is common to all modules (see Figure 1) [27]. If a student subsequently meets the conditions they receive a "Pass after conditions met" or, if they do not met the conditions by the end of the year, the CP is converted into a "Fail". This foundation is consistent with the suggestion, outlined earlier, to make conditional promotions [2,3,17].

2. The development of standards based assessments [28]. As far as possible these are criterion based with text descriptors of levels of achievement and are specified by the relevant module convenor. This helps make the expectations more explicit. Achievement is reported against each different domain of practice based on a variety of assessments, not just for a module as a whole. This means concerns about one attribute, within one module, can be synthesised with concerns of a similar nature in a subsequent attachment. Moreover, mild concerns in one area can be alleviated if a student demonstrates strength in that same area elsewhere. An outline of the assessment programme and process is made known to students at the beginning of each year. This foundation is consistent with the suggestion, outlined earlier, to have transparency and to avoid compensation [3,6,7,14].

3. The use of regular progress meetings throughout each year for module convenors to discuss student progress. This is chaired by the Dean and free exchange of any student issues can be explored. The meetings make use of all available evidence on a student, draw on the collective expertise and judgements of all staff attendees and thereby enhance reliability, validity and defensibility [2,3,6,18,20-22,29]. The Associate Dean for Student Affairs (ADSA) also attends so that relevant information from the student's perspective can be incorporated, and relevant information from the discussions can be conveyed back to the student, thereby assisting in planning subsequent interventions. Relevant information that may help a student's learning can also be conveyed to the convenor of the student's next module. This foundation is consistent with the suggestion, outlined earlier, to make decisions based on accumulated evidence, including qualitative data, and to use multiple judges; it is also consistent with providing feedback and feedforward [2,3,6,17-22].

4. Strengths in one area cannot be used to compensate for deficits in a different area. It is made clear that students are expected to pass all summative assessment components of a module, including aspects of professionalism. While deficits identified early in the year can be remedied later in the year, it is made clear that good performance in one area of practice (e.g. knowledge) cannot compensate for inadequate performance in a different area of practice (e.g. patient interactions). This foundation is consistent with the suggestion, outlined earlier, to avoid compensation [6,7,14].

### Evaluation of the assessment system

The new assessment system was implemented in years 4-6 at all three campuses of the faculty. Over the four years of implementation, the total numbers of problems identified through receipt of a CP were noted for all students in the course, at all campuses.

In addition, at one campus a before and after design was used to compare outcomes from the two most recent years of the system's implementation with the two most recent years prior to implementation. This campus takes approximately one third of the students in years 4-6 of the course. Complete records and text descriptions from all module outcomes were reviewed for these students. The types of problems that arose during each module were clustered into themes by one of the researchers and verified by another. Any discrepancies were resolved by discussion. These themes were quantified alongside any interventions and outcomes for the student.

Comparisons between the old and new system and the likelihoods of passing the year, according to the type of problem, were compared using Chi-square tests and odds ratios. The study was approved by the University of Otago Human Ethics Committee.

## Results

Across the faculty, for students in their final three years of the course, the new system was phased in over four years, culminating in 3539 module results for 701 students by the fourth year of implementation. Each module result could be made up of a number of individual assessments. A failure to achieve the standards in any of these summative assessments leads to a CP for that module. The proportion of module outcome results that were classified as CP slowly increased over that period from 1.4% in the first year, 1.7% in the second year to 4.1% in the third year and 4.1% in the fourth, most recent, year.

For the campus studied in more depth, complete records and text descriptions were analysed. The problems were mostly well outlined and could be clustered into one of the following themes: health, professionalism patient interactions, English as a second language, and poor performance on knowledge tests or assignments. The problems with professionalism occurred in one or more of the following: problems with honesty, problems with reliability, disruptive group behaviour, and/or not maintaining professional boundaries [26].

Table 1 shows the numbers of students (and numbers failing) in each cohort, each class year, and each calendar year before and after the intervention. Because students move from one class year to another in the next calendar year, individual students can be counted more than once.

**Table 1:** Total numbers of students in each cohort prior to and following the intervention

		Class year 4	Class year 5	Class year 6
Pre-intervention	Calendar year 1	60 (0)	62 (8)	62 (0)
	Calendar year 2	71 (2)	66 (1)	58 (0)
Post-intervention	Calendar year 1	86 (4)	70 (6)	60 (2)
	Calendar year 2	80 (4)	86 (8)	65 (0)

Numbers of students failing each year are shown in brackets

Post-intervention, over two years, there were 1516 results available from 447 students, of which 6.5% of the results were classified as CP. In total, 91 of the students (20.4%) had a problem identified during the year that needed consideration (through CP), compared with only 4 students (1.1%) identified prior to the intervention where "borderline", but not CP, was an option (p < 0.001). Under the new system more students failed a year, because of unmet conditions of a CP during the year (20 or 4.5% compared with four or 1.1% under the previous system (p < 0.01)). In contrast, there was no significant difference in the proportions of students failing on the basis of the end of year 5 high stakes examinations only (7/128 under the new system, compared with 4/156 under the old system; p = 0.21), suggesting the standard of students, as measured by knowledge and skills, was similar between cohorts. Health problems affecting academic progress were identified in one student under the previous system compared with four under the new system (p = 0.24).

Although the new system identified significantly more students about whom there were concerns, some patterns emerged that helped eliminate those of little concern and helped identify those at particular risk. Sixty one of the 91 students identified had a problem identified just once and that was unrelated to concerns about professionalism. Only two of those students failed a year (both failed the end of year 5 examinations). The odds ratio for failure for this large subset of students was 0.6 (95% CI 0.13-2.4). In contrast, 38 students had problems identified more than once during the year, of whom 25 failed the year (odds ratio for failure: 18.8 (95% CI 7.7-46.2)).

Table 2 shows the odds ratios for failing a year, under the new system, according to the type of problem. The likelihood of failing was highest for professional issues and lowest for problems with knowledge. Underachievement on an assignment (usually necessitating resubmission of the assignment) was not significantly associated with failing the year.

**Table 2:** Odds ratios for failing a year according to the type of problem

Problem	Total	Passed		Failed	p	Odds ratio for failing
		year		year		(95% confidence intervals)
Professionalism	15	6	40%	9	< 0.0001	17.2 (9.1-33.3)
Attendance & deadlines	8	2	25%	6	< 0.0001	18.2 (10.0-333.3)
Other professional attitudes	7	4	57%	3	0.004	9.0 (3.5-23.3)
Health	4	2	50%	2	0.016	10.1 (3.5-29.4)
Patient interactions	55	42	76%	13	< 0.0001	8.4 (4.0-17.9)
English as a second language	8	5	63%	3	0.006	7.8 (2.9-20.8)
Knowledge tests	34	29	85%	5	0.028	3.2 (1.3-8.0)
Assignments	25	24	96%	1	NS	0.7 (0.1-5.2)
All students	447	423	95%	20		1.0

All students who obtained a CP were brought to the attention of the ADSA and were discussed at student progress meetings. Eighty five students had interviews, 23 of whom also had documentary letters from the ADSA or Dean. Eight were offered assistance with English and 25 had remediation which was either targeted to their needs during the course, or required repeating components of the course over their holiday period.

Of the 22 students who failed a year for reasons other than health, no appeals proceeded beyond students making local enquiries for clarification.

## Discussion

The key components of this system of assessment are setting clear expectations, use of conditional pass (CP), longitudinally monitoring progress and not allowing strengths in one area to compensate for deficits in a different area. This combination has increased our ability to identify more students of concern, has resulted in less "failure to fail", and has increased the detection of (and action on) problems with professionalism.

In screening for potential problems, we aimed to have a sensitive, not necessarily specific, system. Clearly, by identifying around one fifth of the class, the system is more sensitive than specific. However, if we eliminate those students who were identified only once during the year as a cause of potential concern, we eliminate most of the "false positives". It is of relevance to note that the annual 2006 US Clerkship Directors in Internal Medicine survey included a section about how clerkship directors handle struggling third- and fourth-year medical students [20]. Respondents identified 0% to 15% of students as struggling each year during the required core internal medicine clerkship and 0% to 11% of fourth-year students [20]. The current rate of detection among our students of 6.5% is therefore consistent with these findings. It is important to note that students will be counted twice if they had problems in consecutive years, particularly if they failed a year. The rate of detection of *problems *is therefore not an accurate estimate of the prevalence of *students *with problems.

Not all the students who were identified only once would be "false positives". Some may well have learnt from the experience, consistent with assessment being used "for learning", not just "of learning".

One of the unexpected outcomes of this system is the ability to detect, and act on, problems with professionalism. Indeed, problems with professionalism have now become the biggest risk factor for failing a year. We have also noted that significant problems rarely occur in isolation - students at risk of failure have more than one problem (or a problem identified more than once) suggesting that problems in one domain of interest such as professionalism, may also be associated with other problems, such as knowledge. The interaction between these domains of ability is an area worthy of further exploration.

Each of the four components to the system plays an important part and, in combination, attempts to meet the eight requirements of an effective programmatic system of assessment outlined earlier. Standards based assessments [28] contribute to a clearly articulated assessment programme and transparency [3]. Such standards are often text based and this helps define those expectations that are less amenable to measurement in numerical terms.

A central progress committee and the ADSA longitudinally monitor progress and help to accumulate a coherent body of evidence [2,3,6,20-22]. This has several advantages: Firstly, failure of a student is not dependent on one person's decision; instead, the committee provides a collective view based on all available information. There is therefore group accountability for major decisions, rather than decisions and defence resting on one individual. Secondly, multiple sub-threshold problems can be identified and acted on - single episodes of poor performance could be tolerated but if these are seen on several occasions they can be an indicator of a pattern of more serious underlying problems. Having all relevant parties at the same meeting to discuss these can reveal patterns that no single observer could detect. Thirdly, there is peer moderation of decisions that not only increases consistency but also helps in staff professional development [21]. Fourthly, module convenors are more likely to raise concerns in the expectation that they will get advice from their colleagues on the best course of action. Finally, single assessments by single assessors may lack the reliability needed for high stakes decisions. By making decisions that are based on information from a variety of assessments and a variety of assessors, we improve the reliability of the data informing those decisions [2]. Such decision-making procedures are similar to a qualitative approach that continues to accumulate information until saturation is reached and a decision becomes trustworthy and defensible [2]. This also allows us to share problems with the faculty who subsequently teach a student, and thereby assist in remediation.

Reporting on subcomponents of assessment minimises compensation [6,7,14]. For example, there is now less risk that a student with an excellent bedside manner but who is unreliable would not be discussed.

Conditional pass circumvents some of the problems of borderline passes [2,3,17], encourages assessors to describe specific behaviours [3], encourages gathering of more information where there is doubt [2,17-19], assists in providing feedback [2], and automatically creates a paper trail of defensible documentation. Because CP does not result in automatic failure and, in contrast, can actually trigger assistance for a student, faculty are more willing to express concerns. CP is a concept alluded to by others [2] but we have shown how this could be operationalised. We all recognise that some decisions are difficult where a clear pass or clear fail cannot be made confidently [17,19]. Some may refer to these students as borderline, as we did initially. However, we found the term borderline was used inconsistently and was insufficiently specific: it did not necessarily inform the student what to do, it did not help faculty members decide how to help the student and the defensibility was not as robust as we would like. The use of CP has many parallels with employment performance review whereby any underperformance is first made known to the employee and then firm action is possible, and defensible, if the under performance is not remedied. A text-based system, where conditions are put into words, requires more explicit definition of areas of concern than a numerical system. The conditions of a CP not only inform faculty about the extra information that is required, but also tell the students what evidence they need to demonstrate to the faculty that they are safe to proceed. It carries information with respect to specified performances and thereby builds in feedback. There is therefore alignment among the assessment, the learning and the remediation, and there is a paper trail for defensibility [29].

This alignment fits with Stiggins' contrast between 'assessment of learning' and 'assessment for learning' [30]. The crucial distinction is between assessment to determine the status of learning and assessment to promote greater learning [30]. Stiggins states two crucial components: (1) understanding and articulating in advance of teaching the achievement targets that students are to meet; and (2) informing students about those learning goals, in terms that students understand, from the very beginning of the teaching and learning process. Although we need to undertake more work on the educational impact of this system, we suggest that the use of CP might contribute to assessment for learning.

We are aware of one other school that uses an alternative to borderline by using the term "needs assistance" in relation to the welfare and professional attitudes and behaviours of medical students [13]. In that study, the most frequent category was responsibility/reliability (46.7%) followed by participation, respect, relating to others, self appraisal, honesty, integrity and compassion [13]. Our system includes assessment of all attributes, and is not restricted to professional behaviour only. Placing professional attributes on the same level as other academic attributes helps "legitimise" professional behaviour as a core requirement.

The ADSA also has a crucial role as a conduit between the progress committee and the student. At times the ADSA acts as a student advocate by being able to note any relevant health or personal issues while still keeping the details confidential between the ADSA and student; thus separating these details from academic progress issues.

Failing more students was not the aim of this intervention. If it was, then another way to achieve this would have been to just raise the pass threshold for each assessment. However, raising the pass threshold may not fail the right students. Moreover, the aim of our intervention was to detect and assist students, and to be more targeted in our interventions, based on particular needs. This is assisted by acting on aggregated data, not just on results of individual assessments. If some students are unable to be assisted in the time available, then one consequence is a higher rate of failure.

Implementation of any complex system, particularly one that challenges established views on assessment, is not always straightforward. The intervention was implemented earliest in the campus that was evaluated as part of this study. Factors contributing to its successful implementation were likely to be strong support by the campus dean; articulation of clear and consistent messages, backed up by a clear rationale; and incorporation of feedback from staff. This last factor not only helped improve the system and its clarity, but also would have contributed to deeper understanding and ownership of the system by those staff members. It also largely explains the increasing rate of CPs over time.

In evaluating a programme of assessment [31], there are many areas that remain unanswered including: the educational impact of the system, are there adverse effects on students who are identified but who subsequently cause no further concern (the false positives)? What problems are we missing (the false negatives)? Which areas are more remediable than others? What is the interaction between problems with professionalism and problems of a more academic nature? What are the opinions of staff and students on "feedforwarding"?

## Conclusions

In summary, this system of assessment has helped us move towards greater alignment among assessment, learning and remediation. It has facilitated a defensible paper trail. We have found that "joining the dots" between assessment results not only improves defensibility but helps identify previously hard to define concerns, particularly around professionalism.

## Competing interests

The authors declare that they have no competing interests.

## Authors' contributions

TW conceived the study, gathered the data, assisted in data analysis/interpretation and drafted the article. JMcK and MLM assisted in data analysis/interpretation.

Each of the authors, TW, MT, TE, AA, JMcK, MLM, and JR, were involved in analysis and interpretation of data, revising the article critically for important intellectual content; and each read and approved the final version to be published.

## Pre-publication history

The pre-publication history for this paper can be accessed here:

http://www.biomedcentral.com/1472-6920/11/29/prepub
